# Hippo signaling regulates the nuclear behavior and DNA binding times of YAP and TEAD to control transcription

**DOI:** 10.1126/sciadv.adw4974

**Published:** 2025-07-25

**Authors:** Benjamin Kroeger, Samuel A. Manning, Varshini Mohan, Jieqiong Lou, Guizhi Sun, Sara Lamont, Alex J. McCann, Mathias Francois, Jose M. Polo, Elizabeth Hinde, Kieran F. Harvey

**Affiliations:** ^1^ Department of Anatomy and Developmental Biology, Monash University, Clayton, VIC 3800, Australia.; ^2^ Peter MacCallum Cancer Centre, 305 Grattan St, Melbourne, VIC 3000, Australia.; ^3^ School of Physics, University of Melbourne, Parkville, VIC 3010, Australia.; ^4^ Institute for Molecular Bioscience, The University of Queensland, St. Lucia, QLD 4072, Australia.; ^5^ The Charles Perkins Center, School of Medical Sciences, FMH, The University of Sydney, Camperdown, Sydney, NSW 2006, Australia.; ^6^ The Adelaide Centre for Epigenetics and the South Australian Immunogenomics Cancer Institute, Faculty of Health and Medical Sciences, The University of Adelaide, Adelaide, SA, Australia.; ^7^ Department of Biochemistry and Pharmacology, Bio21 Institute, University of Melbourne, Melbourne, VIC 3000, Australia.; ^8^ Sir Peter MacCallum Department of Oncology, The University of Melbourne, Parkville, VIC 3000, Australia.

## Abstract

Over the past two decades, genetic and proteomic screens have identified the Hippo pathway as a complex signaling network that controls tissue growth and human cancer. Despite these advances, our understanding of how Hippo signaling regulates transcription is less clear. To address this, we used live microscopy to study the nuclear behavior of the major Hippo pathway transcription effectors, YAP and TEADs. We reveal that TEADs are a major determinant of YAP DNA binding and nuclear mobility, while YAP minorly influences TEADs. YAP and TEAD1 associate with DNA for longer periods in cells with intrinsically low Hippo pathway activity and upon acute Hippo pathway perturbation. TEAD1 binds the genome on a broad range of timescales, and this is extended substantially in nuclear condensates. Last, a cancer-associated YAP fusion protein exhibits substantially different biophysical behavior than either YAP or TEAD1. Thus, we reveal that Hippo signaling regulates transcription, in part, by influencing the DNA binding times of YAP and TEADs.

## Teaser

Hippo signaling regulates transcription by influencing the DNA dwell times of YAP and TEADs.

## INTRODUCTION

The Hippo pathway is an evolutionarily conserved signaling network that has been implicated in the control of organ growth and regeneration, as well as specific cell fate decisions (*1*–*4*). It operates in a wide range of species from single-celled metazoan ancestors to humans and from the earliest stages of mammalian development through to homeostasis in adults. Defective Hippo signaling also underpins different human diseases, including several cancers (*5*). The Hippo pathway was first discovered and elucidated using *Drosophila melanogaster* genetic screens (*6*–*12*). For simplicity, here, we will use the human nomenclature for Hippo pathway proteins, given our study is based in human cells. The central DNA binding transcription factors in the Hippo pathway are TEA domain transcription factor (TEAD)1-TEAD4. In partnership with the Yes-associated (YAP) and transcriptional co-activator with PDZ binding motif (TAZ) transcription co-activators, TEADs can promote transcription of their target genes (*13*–*16*). Additionally, TEADs can repress transcription with either Vestigial-like family member 4 (VGLL4) or INSM transcriptional repressor 1 (INSM1), both of which can also form a physical complex with TEADs (*17*–*20*). YAP and TAZ activity are regulated by the Hippo pathway core kinase cassette, which, in turn, are regulated by a range of upstream signaling proteins (*1*–*4*). These proteins respond to a variety of cues, including cell-cell adhesion, cell polarity, mechanical forces, and cellular stresses associated with changes in energy availability and osmolarity (*1*–*4*).

To date, the best defined regulatory process in YAP/TAZ-mediated transcription is control of the nucleo-cytoplasmic shuttling rate of YAP and TAZ by the Hippo pathway (*21*). The central Hippo pathway kinases large tumor suppressor kinases 1/2 (LATS1/2) phosphorylate YAP/TAZ on multiple serine residues, which limit the nuclear pool of these proteins and their ability to induce transcription with the TEADs (*22*–*25*). LATS-mediated phosphorylation was initially thought to cause stable sequestration of YAP/TAZ in the cytoplasm, although live imaging studies revealed that the majority of YAP and TAZ continually and rapidly shuttle between the cytoplasm and nucleus, and both Hippo signaling and TEAD abundance modulate the rate at which this happens (*26*–*30*). In addition, Hippo pathway–independent processes can influence YAP/TAZ nucleocytoplasmic shuttling such as nuclear deformation, mechanical forces, and phosphorylation by additional kinases (*27*, *31*, *32*). Furthermore, YAP and TAZ can both undergo liquid-liquid phase separation, and their transcription-promoting activity is higher in YAP/TAZ-enriched nuclear condensates than other regions of the nucleus (*33*–*36*).

What is currently unclear is whether the sole mechanism by which the Hippo pathway regulates transcription is to dictate the YAP/TAZ nucleocytoplasmic shuttling rate and hence nuclear concentration of YAP/TAZ or whether it does so by additional mechanisms. Recently, live microscopy experiments in *Drosophila* tissues revealed that Yki (YAP/TAZ ortholog) overexpression extends Scalloped (TEAD) DNA binding times, while overexpression of the transcription corepressors Tgi (VGLL4) and Nerfin-1 (INSM1) had the opposite effect (*37*). However, it is unknown whether this is also true in human cells or whether changes in Hippo signaling alter YAP/TEAD biophysical behavior such as their mobility and/or their DNA dwell times. Further, alterations in transcription factor DNA dwell times have been associated with both transcription activation and repression. For example, increased DNA dwell times of the Serum Response Factor transcription factor are observed when transcription is elevated (*38*), while extended DNA binding of transcription repressors has been shown to correlate with reduced transcription (*39*).

To investigate the outstanding question of how Hippo signaling regulates transcription, we used high-resolution live imaging modalities to assess the biophysical behavior of YAP and TEAD1 in cells with either intrinsic or chemically induced differences in Hippo pathway activity, as well as in nuclear condensates. Last, to gain insights into the importance of biophysical behavior for transcription factor function, we investigated a cancer-associated variant of YAP (YAP-TFE3) that drives the sarcoma epithelioid hemangioendothelioma (*40*, *41*).

## RESULTS

### YAP and TEAD1 exhibit different biophysical properties in cell nuclei

To investigate the DNA binding properties and molecular mobility of the transcription effectors of the Hippo pathway, YAP and TEADs, we used two microscopy techniques: single-molecule tracking (SMT) and fluorescence fluctuation spectroscopy. To assess YAP and TEAD behavior at single-molecule resolution, we generated plasmids that expressed versions of YAP or TEAD1 fused to HaloTag. HaloTag is derived from a bacterial enzyme that covalently binds to a fluorescently tagged ligand that is bright, highly resistant to photobleaching and is amenable to SMT (*42*). We imaged YAP and TEAD1 at single-molecule resolution and tracked their behavior in the nucleus of living cells, using highly inclined and laminated optical sheet (HILO) microscopy, which is a variant of total internal reflection fluorescence (TIRF) microscopy (*43*). Most experiments were performed using doxycycline-inducible transgenes that were stably integrated into the genome of human MCF10A breast epithelial cells, which are immortalized but non-transformed. Doxycycline was titrated to induce expression of HaloTagged YAP and TEAD1 at endogenous levels or lower (fig. S1, A and B).

**Fig. 1.**
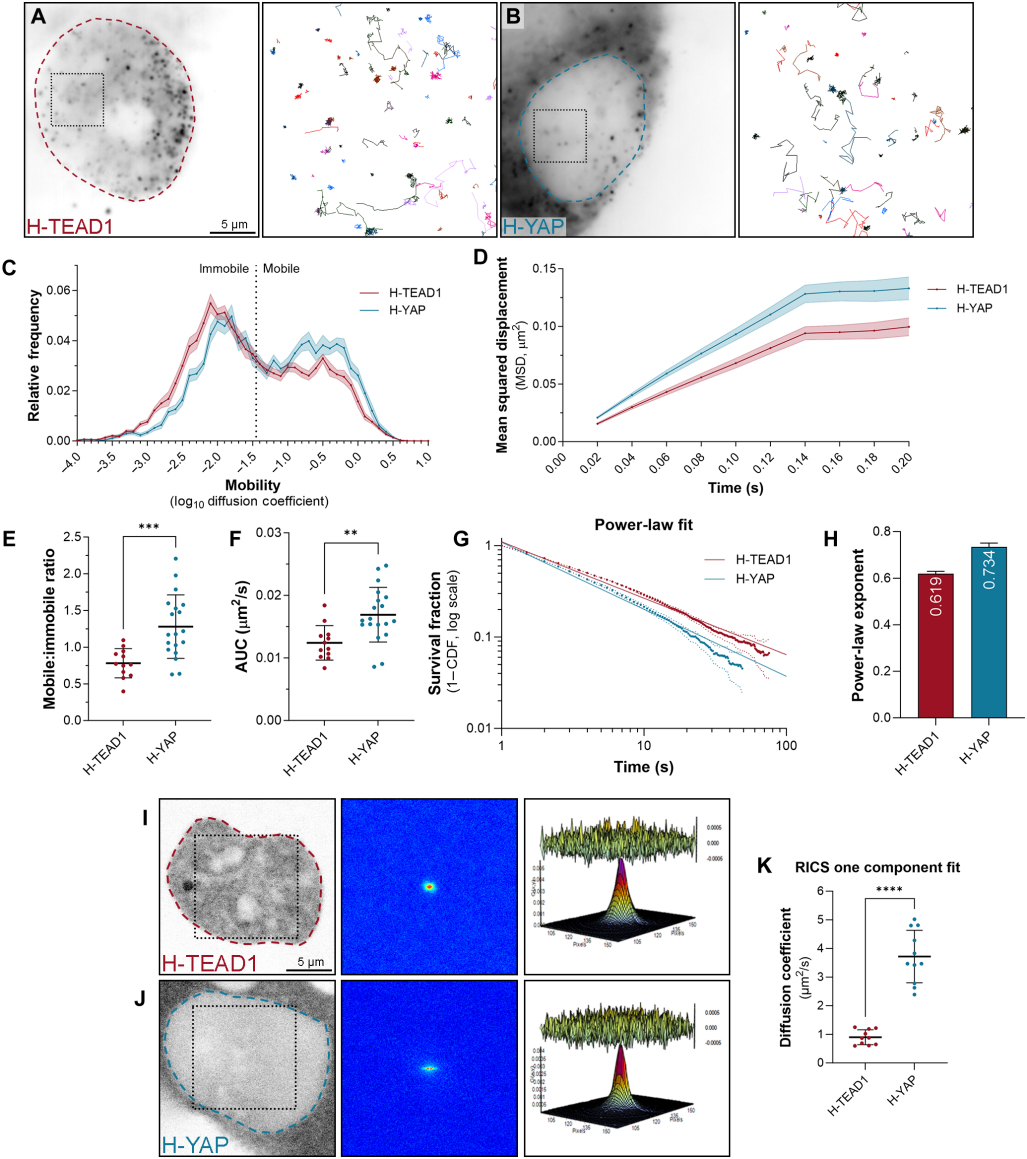
YAP and TEAD1 exhibit different nuclear biophysical properties. (**A** and **B**) Left panels display single MCF10A cells expressing HaloTagged TEAD1 (A) or YAP (B), imaged at single-molecule resolution. Dashed outlines indicate nuclei. Right panels (boxed regions in left panels) show trajectories of TEAD1 or YAP molecules tracked over time. Scale bar indicated. (**C**) Chart of relative frequency of molecule mobilities (log_10_ diffusion coefficient) for TEAD1 (red) and YAP (blue) in MCF10A cell nuclei over time. Data are means ± SEM from 14,053 trajectories from 12 cells (TEAD1) and 6386 trajectories from 19 cells (YAP). (**D**) Chart of mean squared displacement (MSD; square micrometers) of TEAD1 (red) and YAP (blue) molecules in MCF10A cell nuclei over time. Data are means ± SEM. (**E** and **F**) Charts of mobile-to-immobile ratio (E) or area under the curve (AUC) (F) (square micrometers per second) of TEAD1 and YAP molecules in MCF10A cell nuclei. Data are means ± SD; *t* test; **P < 0.01; ***P < 0.001; *n* = 12 (TEAD1) and 19 (YAP). (**G**) Chart of photobleach-corrected survival distribution of TEAD1 or YAP molecules in MCF10A cell nuclei, with power-law fits (solid lines). Dotted lines are ±99% confidence interval (CI). *n* = 61,860 trajectories from 31 cells (TEAD1) and 27,748 trajectories from 20 cells (YAP). CDF, cumulative distribution function. (**H**) Chart of power-law exponents of TEAD1 and YAP in MCF10A cells. Error bars indicate 95% CI. (**I** and **J**) Left panels display TEAD1 (I) or YAP (J) in single MCF10A cells. Scale bar indicated. Boxed region shows analyzed nuclear region. Central panels are RICS correlation functions. Right panels show single-component diffusion model fits of RICS functions; residuals between data and fit shown above. (**K**) Chart of diffusion coefficient (square micrometers per second) of TEAD1 and YAP in MCF10A cell nuclei. Data are means ± SD; *t* test; ****P < 0.0001; *n* = 10 and 11.

Initially, we acquired images of cells every 20 ms (fast SMT) to capture the behavior of most of TEAD1 and YAP molecules (Fig. 1, A and B, and movie S1). Using PALMTracer, we tracked the trajectories of each TEAD1 and YAP molecule and plotted their molecular mobilities (*44*). Both proteins displayed a bimodal mobility distribution comprising mobile and immobile molecules, with YAP in general being more mobile than TEAD1 (Fig. 1C). This was confirmed by calculating the mean squared displacement (MSD) of individual TEAD1 and YAP proteins (Fig. 1D), the ratio of mobile-to-immobile molecules (Fig. 1E), and the area under the curve (AUC; the distance a population of molecules moves in a given time) (Fig. 1F). These different nuclear mobilities likely represent proteins that are freely diffusing (mobile) or bound to DNA (immobile) (*42*). HaloTagged TEAD1 behavior did not vary substantially when expressed at different levels, while HaloTagged YAP became more mobile when expressed ~10-fold higher than endogenous YAP (fig. S1, C to G), highlighting the importance of studying proteins expressed at their native expression levels, which was done for subsequent experiments. We also found that the position of the HaloTag (N terminus or C terminus) did not have a major influence on nuclear TEAD1 or YAP behavior (fig. S2, A to D).

Next, we used SMT to further investigate the immobile pool of TEAD1 and YAP by acquiring images of cells with a longer acquisition duration of 500 ms (slow tracking, movie S2). With this imaging regime, the rapidly moving mobile molecules blur into the background and are not detected, allowing one to better characterize the dynamics of the immobile fraction of molecules, such as their DNA dwell times. The majority of both TEAD1 and YAP molecules displayed short DNA dwell times of around 0.5 to 1 s, and a decreasing number of molecules displayed longer residence times, extending up to more than a minute (Fig. 1G and fig. S2F). Many transcription factors have been reported to bind DNA on two broad timescales, long and short, which are thought to reflect specific and nonspecific DNA binding, respectively (*42*). Accordingly, transcription factor DNA binding times can be calculated using a two-component exponential decay model (*42*). In addition, other studies have reported that transcription factor–DNA binding follows a power-law model, where dwell times occur along a time continuum and are dominated by short binding events (*45*). To test this, we first imaged HaloTagged Histone H2B with slow SMT to determine the bleach rate, given that histones can associate with DNA for extended periods (fig. S2E). We then transformed our SMT data by adjusting for photobleaching and assessed whether the data best fit two-component or three-component exponential models, or a power-law model, using Bayesian information criterion (BIC), which takes into account model complexity and goodness of fit. In general, we found that the DNA binding times reliably fit either the power-law model or a two-component exponential model, so we reported both, in addition to the raw data distribution (Fig. 1G and fig. S2, F to I). The power-law exponent for TEAD1 was 0.619 ± 0.011, and, for YAP, it was 0.734 ± 0.017 (Fig. 1H), indicating that TEAD1 makes more stable contacts with DNA. Consistent with this, biexponential fitting indicated that TEAD1 long and short DNA dwell times (42.1 and 3.4 s) were approximately twice as long as they were for YAP (20.5 and 2.1 s) (fig. S2H). This suggests that YAP binds to TEAD molecules that are already DNA associated or that YAP unbinds TEADs before their release from DNA or a combination of these.

To investigate nuclear TEAD1 and YAP mobility with an independent technique, we used a fluorescence fluctuation spectroscopy approach termed raster image correlation spectroscopy (RICS). RICS allows extraction of intracellular protein mobility from spatial correlation of fluctuations in fluorescence intensity due to fluorescently tagged protein movement throughout confocal laser scanning microscopy time series data acquisition (*46*). In contrast to our SMT studies, molecular mobility via this approach was defined by a single component, which represents an ensemble of all the detected molecular behaviors. Consistent with our SMT studies, YAP was substantially more mobile than TEAD1 (Fig. 1, I to K). Notably, the diffusion coefficients of HaloTagged TEAD1 and YAP (1 and 3.6 μm^2^/s, respectively) (Fig. 1K) were very similar to our previous observations of endogenously tagged versions of the *Drosophila* orthologs of these proteins in vivo (Scalloped, 1.2 μm^2^/s; and Yorkie, 2.9 μm^2^/s) (*37*).

### TEAD1’s ability to bind DNA is the primary determinant of its nuclear behavior

**Fig. 2.**
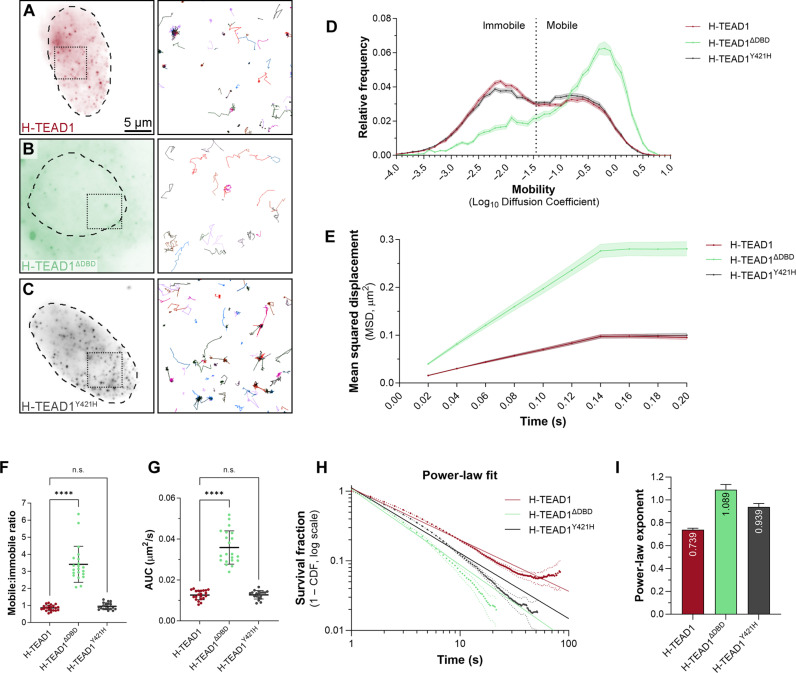
TEAD1’s ability to bind DNA, not YAP, is the primary determinant of its nuclear behavior. (**A** to **C**) Left panels are average intensity projection images of single HeLa cells expressing HaloTagged TEAD1 proteins; wild-type (A), TEA DNA binding domain mutant (B), or TEAD^Y421H^ (C), imaged at single-molecule resolution by HILO. Dashed outlines indicate the nucleus. Right panels show the trajectories of individual TEAD1 molecules tracked over time. Boxed regions in cell images indicate the area corresponding to trajectories. Scale bar is indicated. (**D**) Chart of relative frequency of molecule mobilities (log_10_ diffusion coefficient) of TEAD1 (red), TEAD1^ΔDBD^ (green), and TEAD1^Y421H^ (gray) molecules in HeLa cell nuclei over time. Data are presented as the means ± SEM. Mobile and immobile fractions are indicated. Data are from 73,348 trajectories from 21 cells for TEAD1, 17,369 trajectories from 21 cells for TEAD1^ΔDBD^, and 67,321 trajectories from 19 cells for TEAD1^Y421H^. (**E**) Chart of MSD (square micrometers) of TEAD1 (wild-type and mutant) molecules in HeLa cell nuclei over time. Data are presented as means ± SEM. (**F** and **G**) Charts of mobile-to-immobile ratio (F) or AUC (G) (square micrometers per second) of TEAD1, TEAD1^ΔDBD^, or TEAD1^Y421H^ in HeLa cell nuclei. Data are presented as means ± SD; *P* values were obtained using a Brown-Forsythe and Welch analysis of variance (ANOVA) with Dunnett’s T3 multiple comparisons test; ****P < 0.0001; n.s., not significant; *n* = 21 cells (TEAD1), 21 cells (TEAD1^ΔDBD^), and 19 cells (TEAD1^Y421H^). (**H**) Chart of photobleach-corrected survival distribution of TEAD1 (wild-type or mutant) molecules in the nucleus of HeLa cells, with power-law fits (solid lines). Dotted lines are 99% CI. *n* = 202,613 trajectories from 26 cells (TEAD1), 38,766 trajectories from 22 cells (TEAD1^ΔDBD^), and 83,778 trajectories from 21 cells (TEAD1^Y421H^). (**I**) Chart of power-law exponents of TEAD1 proteins. Error bars indicate 95% CI.

Most proteins move within cells via diffusion, and this can be influenced by physical interactions that they make with other proteins or cellular constituents such as nucleic acids (*47*). Given that TEAD1 can bind both DNA via its TEA domain and proteins like YAP and TAZ, we investigated the relative contributions that these make to its nuclear behavior. To do this, we expressed HaloTagged versions of either wild-type TEAD1, TEAD1 that lacks the TEA domain (TEAD1^ΔDBD^), or TEAD1^Y421H^ [YAP and TEAD1 interact predominantly via a hydrophobic bond between a single–amino acid pair (TEAD1 Y421–YAP S94), and mutation of either of these residues compromises their ability to promote transcription] (*16*, *48*). The importance of the YAP-TEAD interaction is further emphasized by the fact that the human genetic disease Sveinsson’s chorioretinal atrophy is caused by the TEAD1^Y421H^ point mutation (*49*). Using fast SMT, we found that TEAD1^ΔDBD^ was substantially more mobile than either TEAD1 or TEAD1^Y421H^, which displayed very similar mobilities (Fig. 2, A to G, and movie S3). This indicates that DNA binding, but not YAP/TAZ binding, is a major determinant of the nuclear mobility of TEAD1. Consistent with published studies, TEAD1’s DNA binding domain also promoted its nuclear localization, while YAP/TAZ binding did not (Fig. 2, A and B). We then used slow SMT to examine the immobile fractions of these different TEAD1 variants. The power-law exponent for TEAD1 was 0.739 ± 0.014, and, for TEAD1^ΔDBD^, it was 1.089 ± 0.046 (Fig. 2I), indicating that TEAD1 makes far more stable contacts with DNA than TEAD1^ΔDBD^, as expected. The TEAD1^Y421H^ power-law exponent was 0.939 ± 0.029, indicating that, when TEAD1 is incapable of binding to YAP/TAZ, it binds DNA less stably (Fig. 2I). Consistent with this, biexponential fitting indicated that long and short DNA dwell times were reduced in both the TEAD1^ΔDBD^ and TEAD1^Y421H^ variants (TEAD1, 85.1 and 3.6 s; TEAD1^ΔDBD^, 6.7 and 1.2 s; and TEAD1^Y421H^, 14.5 and 2.1 s) (fig. S3, A to D).

### TEADs have a major influence on YAP’s nuclear behavior

**Fig. 3.**
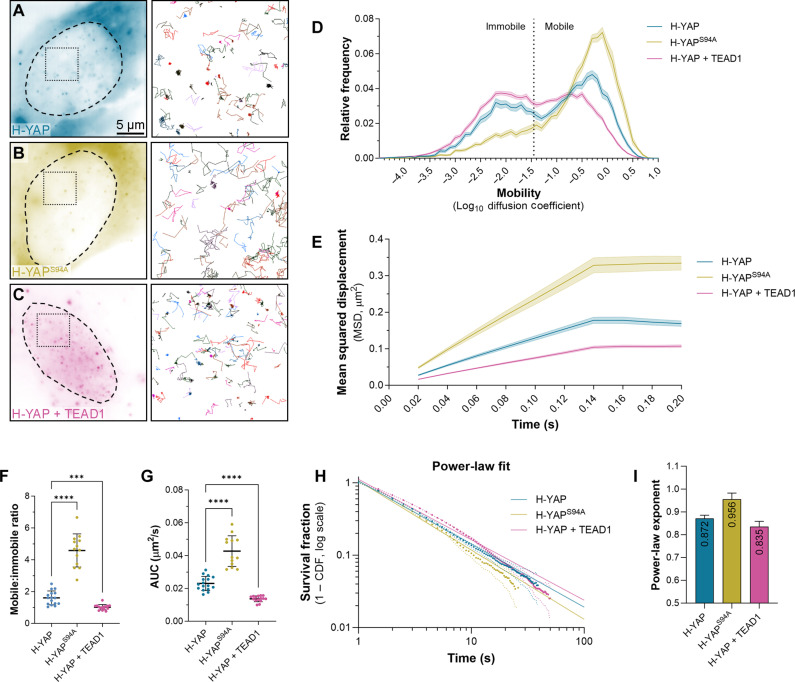
TEADs have a major influence on YAP’s nuclear behavior. (**A** to **C**) Left panels show single HeLa cells expressing HaloTagged YAP proteins; wild-type (A), YAP^S94A^ (B), and YAP with overexpressed TEAD1 (C), imaged at single-molecule resolution. Dashed outlines indicate nuclei. Right panels (boxed regions in left panels) show trajectories of individual molecules tracked over time. Scale bar indicated. (**D**) Chart of relative frequency of molecule mobilities (log_10_ diffusion coefficient) of YAP (blue), YAP^S94A^ (olive), and YAP with overexpressed TEAD1 (pink) molecules in HeLa cell nuclei over time. Data are presented as means ± SEM. Data are from 14,648 trajectories from 16 cells (YAP), 13,785 trajectories from 13 cells (YAP^S94A^), and 52,442 trajectories from 15 cells (YAP with overexpressed TEAD1). (**E**) Chart showing MSD (square micrometers) of YAP (wild-type and mutant) molecules in HeLa cell nuclei over time. Data are presented as means ± SEM. (**F** and **G**) Charts of mobile-to-immobile ratio (F) or AUC (G) (square micrometers per second) of YAP, YAP^S94A^, or YAP with overexpressed TEAD1 in HeLa cell nuclei. Data are presented as means ± SD; *P* values were obtained using a Brown-Forsythe and Welch ANOVA with Dunnett’s T3 multiple comparisons test; ***P < 0.001; ****P < 0.0001; n.s., not significant; *n* = 16 cells (YAP), 13 cells (YAP^S94A^), and 15 cells (YAP with overexpressed TEAD1). (**H**) Chart showing photobleach-corrected survival distribution of YAP (wild-type or mutant) molecules in HeLa cell nuclei, with power-law fits (solid lines). Dotted lines are 99% CI. *n* = 62,126 trajectories from 24 cells (YAP), 33,571 trajectories from 13 cells (YAP^S94A^), and 126,127 trajectories from 16 cells (YAP with overexpressed TEAD1). (**I**) Chart showing power-law exponents of TEAD1 proteins. Error bars indicate 95% CI.

We next examined how TEADs affect YAP nuclear behavior and chromatin association by studying a mutant YAP protein (YAP^S94A^, which cannot bind to TEADs) (*16*), as well as the impact of TEAD1 overexpression on YAP. HeLa cells were used for these experiments because of their high transfection efficiency; only cells with similarly low protein expression were imaged. Fast SMT revealed that TEADs have a major influence on YAP behavior; while YAP exhibited both mobile and immobile behavior in the nucleus, YAP^S94A^ was almost entirely mobile (Fig. 3, A to G). RICS also showed that nuclear YAP^S94A^ was far more mobile than YAP (fig. S4A). In addition, TEAD1 overexpression substantially decreased the overall nuclear mobility of YAP by SMT (Fig. 3, A to G) and transformed its overall mobility distribution to almost match TEAD1 (compare to Figs. 1 and 2). These phenotypes were evident with every quantitative analysis we performed, i.e., mobility, MSD, AUC, and mobile-to-immobile ratio (Fig. 3, D to G).

Next, we examined the impact of TEADs on YAP chromatin association, using slow SMT. The power-law exponent for YAP was 0.872 ± 0.014 (Fig. 3, H and I), while, for YAP^S94A^, it was 0.956 ± 0.026, indicating less stable DNA binding. In comparison to YAP alone, overexpression of TEAD1 slightly reduced YAP’s power-law exponent to 0.835 ± 0.023. Two-component fitting of DNA dwell times showed that YAP^S94A^ had reduced short and long DNA binding times relative to YAP, but overexpression of TEAD1 had no major impact on YAP’s DNA binding times (YAP, 19 and 1.8 s; YAP^S94A^, 11.1 and 1.5 s; and YAP + TEAD1, 17.8 and 2.2 s). Instead, TEAD overexpression increased the fraction of longer-lived YAP DNA binding times from ~15 to 20% (fig. S4, B to E). Together with the fast-tracking results, these data show that TEADs are a major regulator of YAP’s nuclear mobility, and, consistent with numerous previous studies, TEADs facilitate YAP’s association with the genome.

### Hippo signaling limits the DNA dwell times of YAP and TEAD1

**Fig. 4.**
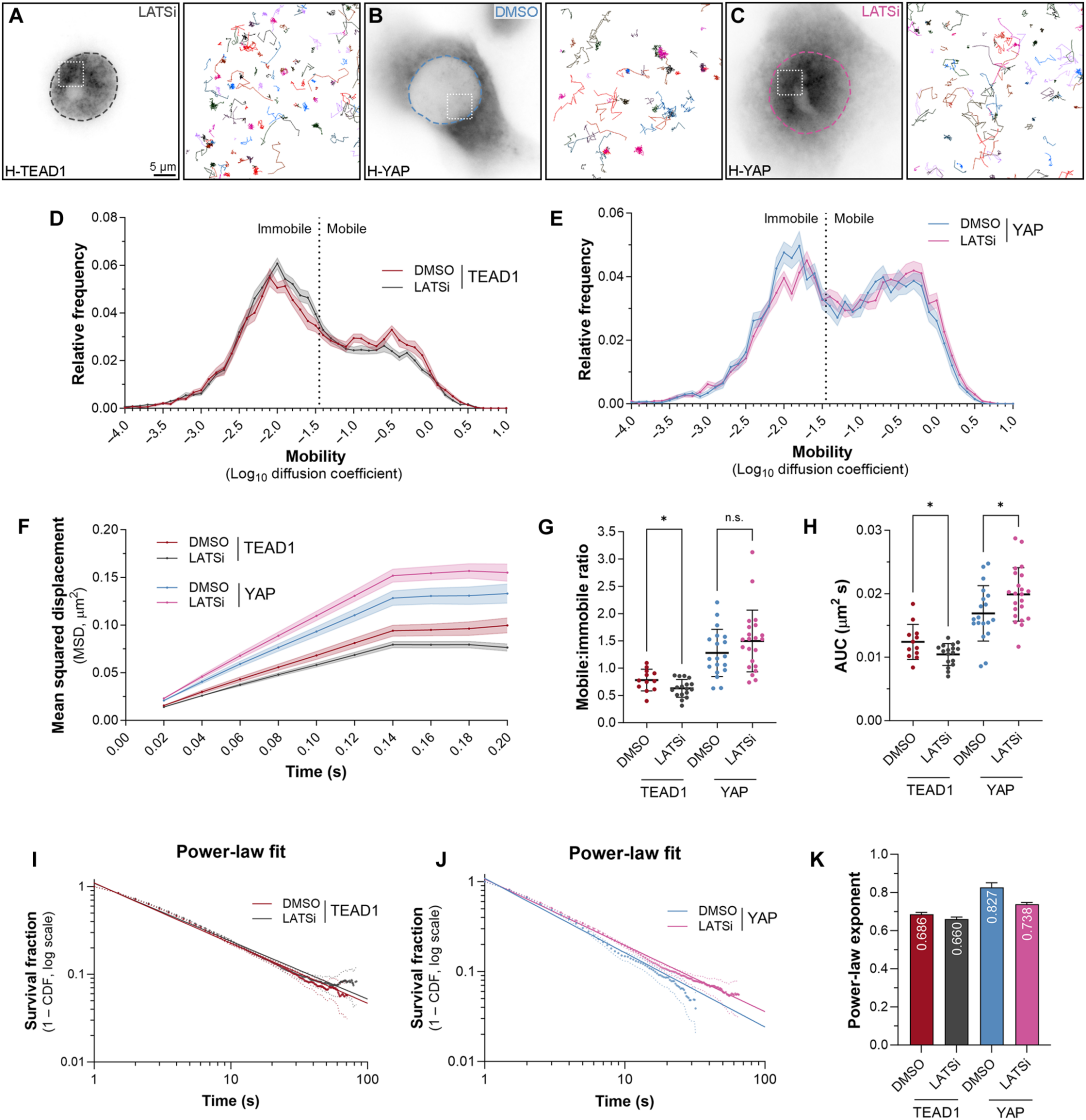
Hippo signaling limits YAP and TEAD1 DNA binding times. (**A** to **C**) Left panels show single MCF10A cells expressing HaloTagged TEAD1 (A) or YAP (B and C), treated with 3 μM LATSi (A and B) or dimethyl sulfoxide (DMSO) (B). Dashed outlines indicate nuclei. Right panels (boxed regions in left panels) show trajectories of individual molecules tracked over time. Scale bar indicated. (**D** and **E**) Chart of relative frequency of molecule mobilities (log_10_ diffusion coefficient) of TEAD1 (D) or YAP (E) in MCF10A cell nuclei over time (treated with DMSO or LATSi). Data are presented as means ± SEM. Data are from 14,053 trajectories from 12 cells (TEAD1, DMSO), 20,093 trajectories from 17 cells (TEAD1, LATSi), 6386 trajectories from 19 cells (YAP, DMSO), and 10,752 trajectories from 22 cells (YAP, LATSi). (**F**) Chart of MSD (square micrometers) of TEAD1 or YAP molecules in MCF10A cell nuclei over time (treated with DMSO or LATSi). Data are presented as means ± SEM. (**G** and **H**) Charts of mobile-to-immobile ratio (G) or AUC (H) (square micrometers per second) of TEAD1 and YAP molecules in MCF10A cell nuclei (treated with DMSO or LATSi). Data are presented as means ± SD; *P < 0.05 (unpaired *t* test); n.s., not significant; *n* = 12 and 17 cells (TEAD1) and *n* = 19 and 22 cells (YAP). (**I** and **J**) Chart of photobleach-corrected survival distribution of TEAD1 or YAP molecules in MCF10A cell nuclei, with power-law fits (solid lines, treated with DMSO or LATSi). Dotted lines are 99% CI. *n* = 54,886 trajectories from 21 cells (TEAD1, DMSO), 68,992 trajectories from 17 cells (TEAD1, LATSi), 14,104 trajectories from 19 cells (YAP, DMSO), and 46,056 trajectories from 22 cells (YAP, LATSi). (**K**) Chart of power-law exponents of YAP and TEAD1 in MCF10A cells (DMSO or LATSi). Error bars indicate 95% CI.

How signaling pathways affect the biophysical behavior and DNA binding of their downstream transcription factors has only begun to be explored. Exceptions include the Notch and Glucocorticoid pathways, which both stimulate transcription by inducing an increase in dwell time of their relative transcription factors (*50*, *51*). The best characterized way in which Hippo signaling controls transcription is by reducing the nuclear pool of YAP by phosphorylating it (*1*–*4*). To study the impact of Hippo signaling on YAP and TEAD1 nuclear behaviors and DNA binding, we used recently developed LATS inhibitors (LATSi), which rapidly suppress LATS1/2-dependent phosphorylation of YAP (*52*). MCF10A cells were treated with LATSi or dimethyl sulfoxide (DMSO) for 2 hours and YAP and TEAD1 imaged by SMT, as we observed robust loss of YAP-S127 phosphorylation (fig. S5A). Consistent with previous findings that LATS1/2 limit YAP’s nuclear access (*24*, *25*), we observed a substantial increase in nuclear Halo-YAP following LATSi treatment, while TEAD1 localization was unaffected (Fig. 4, A to C). Fast SMT revealed that TEAD1 nuclear mobility was decreased slightly following LATS1/2 inhibition (Fig. 4D). By contrast, YAP mobility was slightly increased by LATS1/2 inhibition (Fig. 4E). These changes were evident when assessing MSD and AUC (Fig. 4, F and H). A notable change was also observed in the mobile-to-immobile ratio of TEAD1; for YAP, this trended toward significance (*P* = 0.169) (Fig. 4G). When we performed the same experiments using RICS, we were unable to detect any substantial differences in YAP or TEAD1 mobility when LATS activity was inhibited (fig. S5B).

We next examined whether blocking Hippo signaling affected YAP and TEAD1 chromatin association, by performing slow SMT in MCF10A cells treated with LATSi for 2 hours. The power-law exponents of both and YAP and TEAD1 were lower in LATSi-treated cells, indicating that their DNA dwell times were extended (Fig. 4, I to K). We then extracted long and short DNA dwell times from the two-component exponential fit (fig. S5, D to F). These revealed that both YAP and TEAD1 long DNA binding times doubled after LATSi treatment: YAP increased from 18.3 to 41.4 s, while TEAD1 went from 42.2 to 82.5 s. Similarly, short-lived DNA binding times were also slightly increased. In contrast, both TEAD1 and YAP had a slightly reduced fraction of long-lived binding events (fig. S5G). Therefore, Hippo signaling normally limits the amount of nuclear YAP and the time that both YAP and TEAD1 associate with the genome.

### YAP and TEAD1 associate with DNA for extended periods in cells with intrinsically low Hippo signaling

To investigate the impact of Hippo signaling in an independent fashion and a more physiological setting, we leveraged knowledge of Hippo signaling in the early mammalian embryo, where it is essential for the very first cell fate choice, i.e., trophectoderm versus inner cell mass (*53*). Hippo signaling is high in the inner cell mass, which goes on to develop the embryo, while it is low in the trophectoderm, which forms the placenta (*53*). Differential Hippo signaling in these two juxtaposed cell types results in high YAP/TEAD activity in the trophectoderm and low YAP/TEAD activity in the inner cell mass (*53*). As such, it is an ideal biological system to explore YAP/TEAD dynamics. However, the physical dimensions of the early mammalian embryo were not compatible for reliable single-molecule imaging as HILO imaging substantially deteriorates the further the sample is from the coverslip. Therefore, to image YAP and TEAD1 at single-molecule resolution in living cells resembling the early embryo, we used two well-known two-dimensional (2D) cellular models: induced pluripotent stem cells (iPSCs), to model the epiblast, and induced trophoblast stem cells (iTSCs), to model the trophoblasts (*54*, *55*). To do this, we stably expressed Dox-inducible Halo-YAP and Halo-TEAD1 in iPSCs or iTSCs. Both YAP and TEAD1 were nuclear in both cell types when grown in monocultures, as determined with YAP antibodies (fig. S6A). However, when these cells were grown as iTSC/iPSC cocultures, iPSCs grew together in spheres, where YAP was cytoplasmic, as it is in the inner cell mass of the early embryo (fig. S6, B and D). By contrast, YAP remained nuclear in iTSCs that surround the iPSC islands, as it is in the trophectoderm (fig. S6, B and C). As such, this iTSC/iPSC coculture system provided the ideal setting to study YAP and TEAD1 behavior at single-molecule resolution in cells with intrinsic differences in Hippo signaling.

**Fig. 5.**
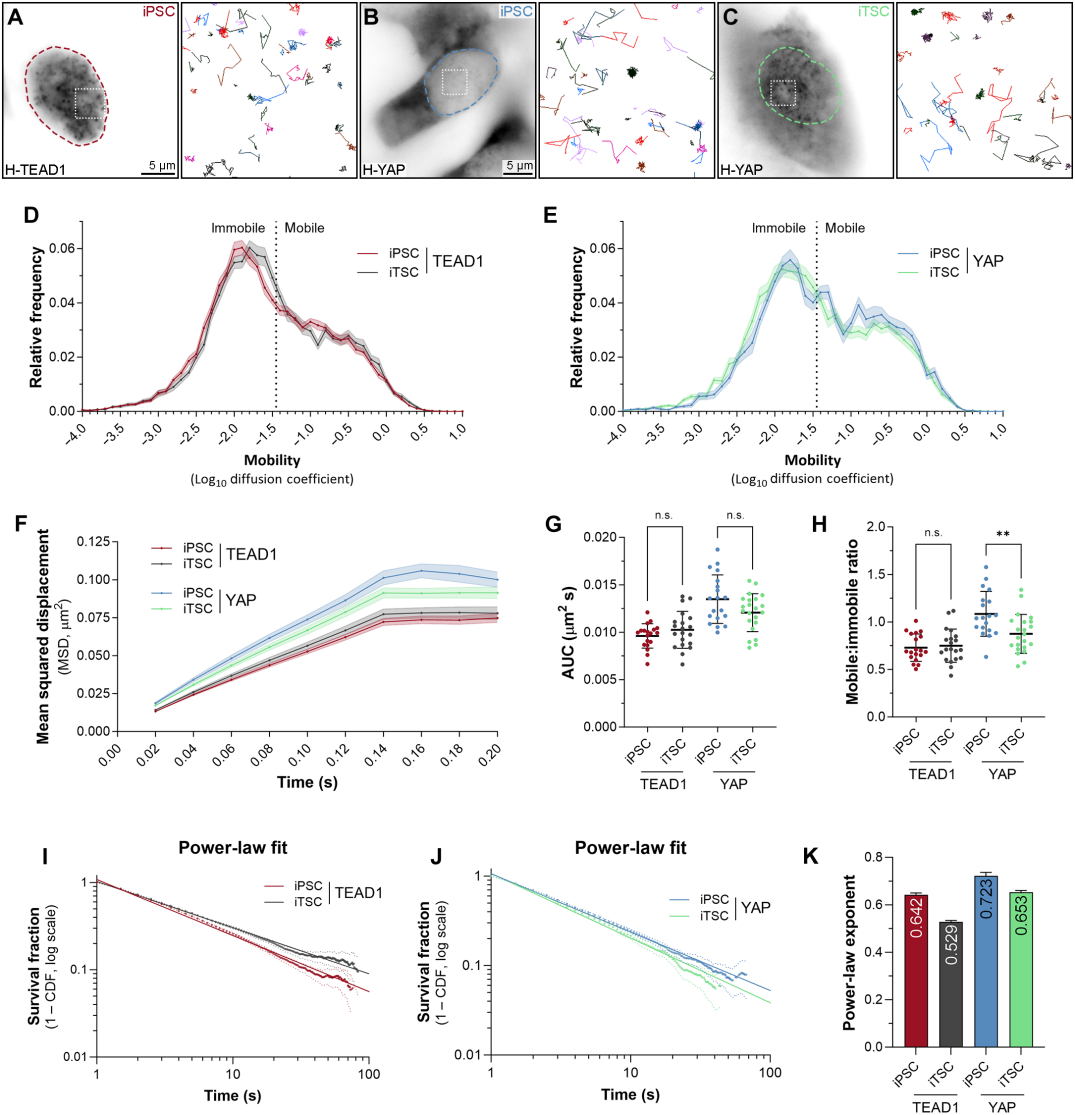
YAP and TEAD1 bind DNA longer in cells with intrinsically high YAP/TEAD activity. (**A** to **C**) Left panels are single iPSC or iTSC expressing HaloTagged TEAD1 (A) or YAP (B and C). Dashed outlines indicate nuclei. Right panels (boxed regions in left panels) show trajectories of individual molecules tracked over time. Scale bars indicated. (**D** and **E**) Chart of relative frequency of molecule mobilities (log_10_ diffusion coefficient) of TEAD1 (D) or YAP (E) in iPSC or iTSC nuclei. Data are presented as means ± SEM. Mobile and immobile fractions are indicated. Data are from 17,036 trajectories from 19 cells for TEAD1 (iPSC), 17,257 trajectories from 20 cells for TEAD1 (iTSC), 6874 trajectories from 19 cells for YAP (iPSC), and 23,382 trajectories from 22 cells for YAP (iTSC). (**F**) Chart of MSD (square micrometers) of TEAD1 or YAP molecules in iPSC or iTSC nuclei over time. Data are presented as the means ± SEM. (**G** and **H**) Charts of mobile-to-immobile ratio (G) or AUC (H) (square micrometers per second) of TEAD1 and YAP molecules in iPSC or iTSC nuclei. Data are presented as means ± SD; **P < 0.01 (unpaired *t* test); n.s., not significant; *n* = 19 and 20 cells (TEAD1) and *n* = 19 and 22 cells (YAP) (iPSC and iTSC, respectively). (**I** and **J**) Chart of photobleach-corrected survival distribution of TEAD1 or YAP molecules in cocultured iPSC or iTSC nuclei, with power-law fits (solid lines). Dotted lines are 99% CI. *n* = 47,191 trajectories from 22 cells (TEAD1, iPSC), 42,339 trajectories from 22 cells (TEAD1, iTSC), 13,381 trajectories from 25 cells (YAP, iPSC), and 26,931 trajectories from 23 cells (YAP, iTSC). (**K**) Chart of power-law exponents of YAP and TEAD1 in cocultured iPSC and iTSC nuclei. Error bars indicate 95% CI.

Fast SMT revealed that TEAD1’s nuclear mobility was indistinguishable in both iPSC and iTSC (Fig. 5, D to H). Although YAP was more nuclear iTSCs than iPSCs when grown in coculture, YAP’s nuclear dynamics were also very similar in both cell types (Fig. 5E). YAP mobility, as assessed by MSD and AUC, did not differ significantly, while the mobile-to-immobile ratio of YAP was slightly lower in iTSC (Fig. 5, F to H). Next, we assessed the DNA association dynamics of TEAD1 and YAP in these two cell types, using slow SMT. The TEAD1 power-law exponent was 0.642 ± 0.008 in iPSC and 0.529 ± 0.005 in iTSC, indicating that TEAD1 binds DNA substantially longer in iTSC than iPSC (Fig. 5, I to K). This was also evidenced when fitting the data to a two-component exponential decay model, where the long binding time of TEAD1 increased from ~50 to ~90.9 s (fig. S7, B and D). Similar results were observed for YAP, which had a power-law exponent of 0.723 ± 0.014 in iPSC and 0.653 ± 0.007 in iTSC (Fig. 5, J and I). When fitted to a two-component exponential decay model, the YAP long binding time increased from ~20.3 to ~40.9 s (fig. S7, C and D). Slight increases were also observed for both YAP and TEAD1 short dwell times, and minor changes in the long and short binding fractions (fig. S7, D and E). Therefore, both TEAD1 and YAP bind chromatin for longer in cells with lower intrinsic Hippo pathway activity and where YAP is more nuclear.

### TEAD1 mobility is impaired in nuclear condensates

**Fig. 6.**
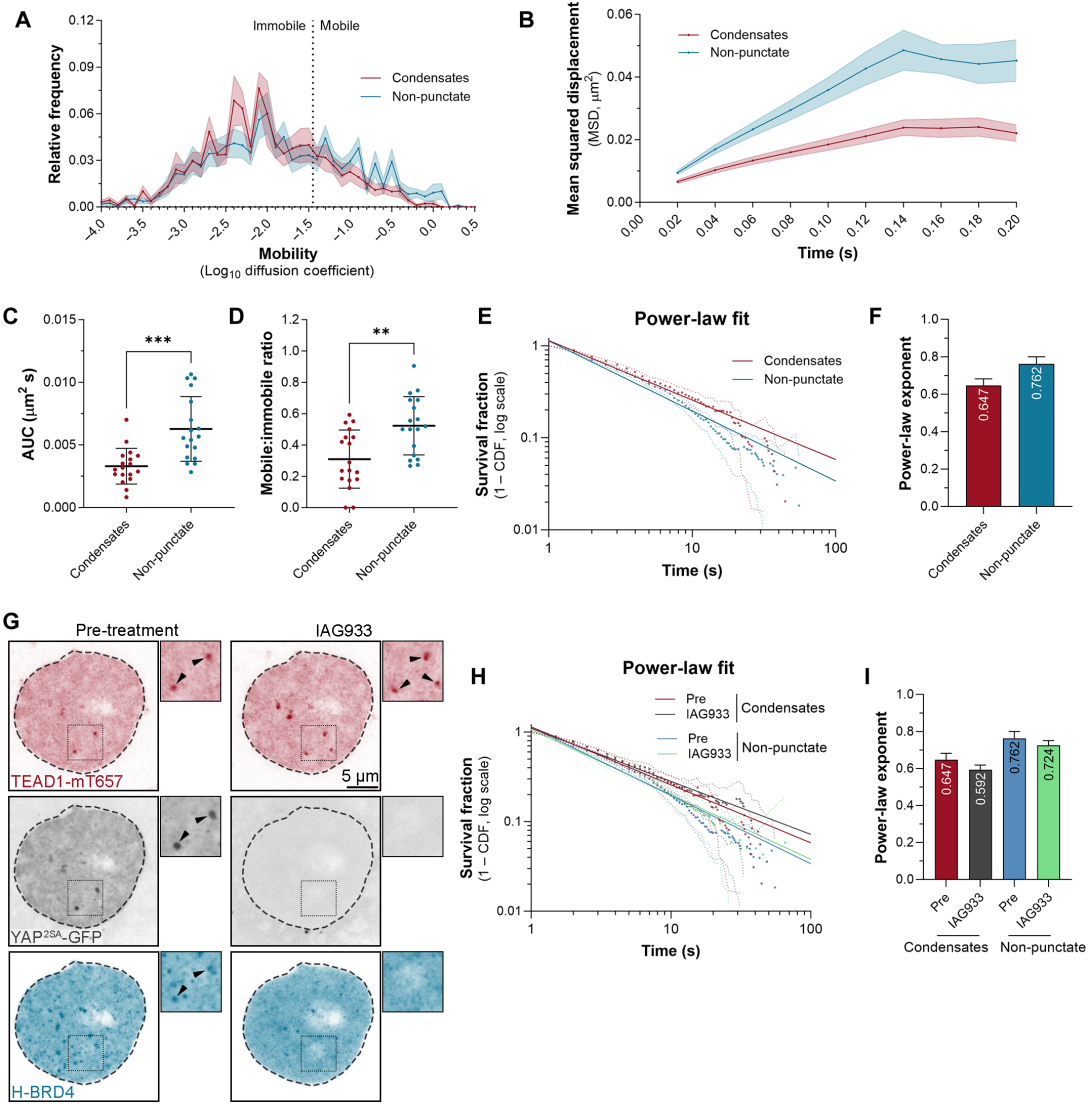
TEAD1 exhibits reduced mobility and extended DNA binding behavior in nuclear condensates. (**A**) Chart of relative frequency of molecule mobilities (log_10_ diffusion coefficient) of TEAD1 in MCF10A cell nuclear condensates and non-punctate nuclear regions (*n* = 1247 and 955 trajectories from 18 cells). Data is means ± SEM. (**B**) Chart of MSD (square micrometers) of TEAD1 in MCF10A cell nuclear condensates and non-punctate nuclear regions, over time. Data is means ± SEM. (**C** and **D**) Charts showing mobile-to-immobile ratio (C) or AUC (D) (square micrometers per second) of TEAD1 in MCF10A cell nuclear condensates and non-punctate nuclear regions. Data is means ± SD; **P < 0.01; ***P < 0.001; unpaired *t* tests; *n* = 18 cells. (**E**) Chart of photobleach-corrected survival distribution of TEAD1 molecules in MCF10A cell nuclear condensates and non-punctate nuclear regions (*n* = 1080 and 2628 trajectories from 14 cells), with power-law fits (solid lines). Dotted lines are 99% CI. (**F**) Chart of power-law exponents of TEAD1 in MCF10A cell nuclear condensates and non-punctate nuclear regions. Error bars indicate 95% CI. (**G**) A HeLa cell expressing TEAD1-mT657 (red), YAP^2SA^-EGFP (gray), and HaloTagged-BRD4 (turquoise) pre- and posttreatment with 3 μM IAG933 for ~1 min. Dashed outlines indicate the nucleus. Black arrowheads indicate condensates having TEAD1, YAP^2SA^, and BRD4 pretreatment and TEAD1 post–IAG933 treatment. Zoomed images of boxed regions are shown. Scale bar indicated. (**H**) Chart showing photobleach-corrected survival distribution TEAD1 in MCF10A cell nuclear condensates and non-punctate nuclear regions, pre- and posttreatment with 3 μM IAG933 for 30 min, with power-law fits (solid lines). Dotted lines are 99% CI. Trajectory numbers were: 1080, 2628, 672, and 1465 from 14 cells. (**I**) Chart of power-law exponents of TEAD1 in MCF10A cell nuclear condensates and non-punctate nuclear regions, pre- and posttreatment with 3 μM IAG933 for 30 min. Error bars indicate 95% CI.

Throughout the course of imaging, we noted that DNA binding times occurred on a broad continuum. Therefore, we explored whether it was heterogeneous across the nucleus, particularly given reports that both YAP and TAZ form nuclear condensates (*33*–*36*), which are membraneless subdomains where these proteins are concentrated. YAP and TAZ condensates are also enriched for TEAD transcription factors and chromatin-regulatory proteins like BRD4 that promote transcription (*33*–*36*). We also observed these condensates in HeLa cells expressing both YAP–green fluorescent protein (GFP) and Halo-TEAD1 (movie S4). YAP exhibits reduced mobility in nuclear condensates (*34*), and, although TEAD is present in these condensates, its behavior in them has not been addressed. To investigate this, we performed SMT in Halo-TEAD1–transduced MCF10A cells and quantified TEAD1 behavior in condensates compared with non-punctate regions of nuclei (fig. S8A). In nuclear condensates, TEAD1 was substantially less mobile, as determined by fast SMT (Fig. 6, A to D). To further address whether the observed continuum of TEAD1’s DNA binding durations are affected by subnuclear domains, we performed slow SMT and found that TEAD1’s DNA binding behavior best fit a power-law model in both condensates and non-punctate nuclear regions (Fig. 6E). The TEAD1 power-law exponent was 0.647 ± 0.055 in condensates, compared to 0.762 ± 0.038 in non-punctate regions, indicating that TEAD1 dwell times were extended in condensates (Fig. 6F). Biexponential fitting, on the other hand, indicated that the fraction of long-lived TEAD1 DNA binding events was greater in condensates, with ~35% of tracks being long, relative to ~23% in non-puncta, while long DNA binding times were similar (both ~17 s) (fig. S8, C to G).

To further investigate the nature of TEAD condensates, we assessed the localization of proteins that promote TEAD-regulated transcription, i.e., YAP^2SA^ (a hyperactive YAP allele), BRD4, which bind acetylated histones, and MED1, from the mediator complex (*56*, *57*). These proteins were investigated in cells before and after treatment with a compound (IAG933) that disrupts the YAP/TEAD protein-protein interaction and alters their genome occupancy at target sites (*58*). Consistent with the idea that TEAD condensates can be sites of active transcription, YAP^2SA^, BRD4 and MED1 were all enriched in TEAD1 condensates in untreated cells (Fig. 6G; fig. S9, A to H; and movie S5). Notably, within 1 min of IAG933 treatment, YAP^2SA^, BRD4, and MED1 were all evicted from TEAD1 condensates (Fig. 6G; fig. S9, A to H; and movie S5). In stark contrast, TEAD1’s nuclear localization was unaffected and, unexpectedly, TEAD1 condensates were unperturbed or increased in size and number upon IAG933 treatment (Fig. 6G, fig. S8D, and movie S5). These data indicate that TEAD condensates in these experiments are sites of active transcription and that this depends on the YAP/TEAD interaction as YAP, BRD4, and MED1 localization to TEAD condensates was potently and rapidly disrupted by IAG933. Subsequently, we performed slow SMT on TEAD1 in nuclear condensates pre- and post-addition of IAG933 in live MCF10A cells to assess any effects on its DNA binding behavior. Power-law models indicated that TEAD1 DNA binding times were extended following IAG933 treatment, in both condensates and in non-punctate nuclear regions (Fig. 6, H and I). Biexponential fitting agreed with this impact on condensate-associated TEAD1, as long DNA binding times in particular were extended following IAG933 treatment (16.7 s in condensates pre–IAG933 treatment and 30.4 s post–IAG933 treatment) (fig. S8, E to G).

### A cancer-associated YAP fusion protein displays different nuclear behavior than YAP

**Fig. 7.**
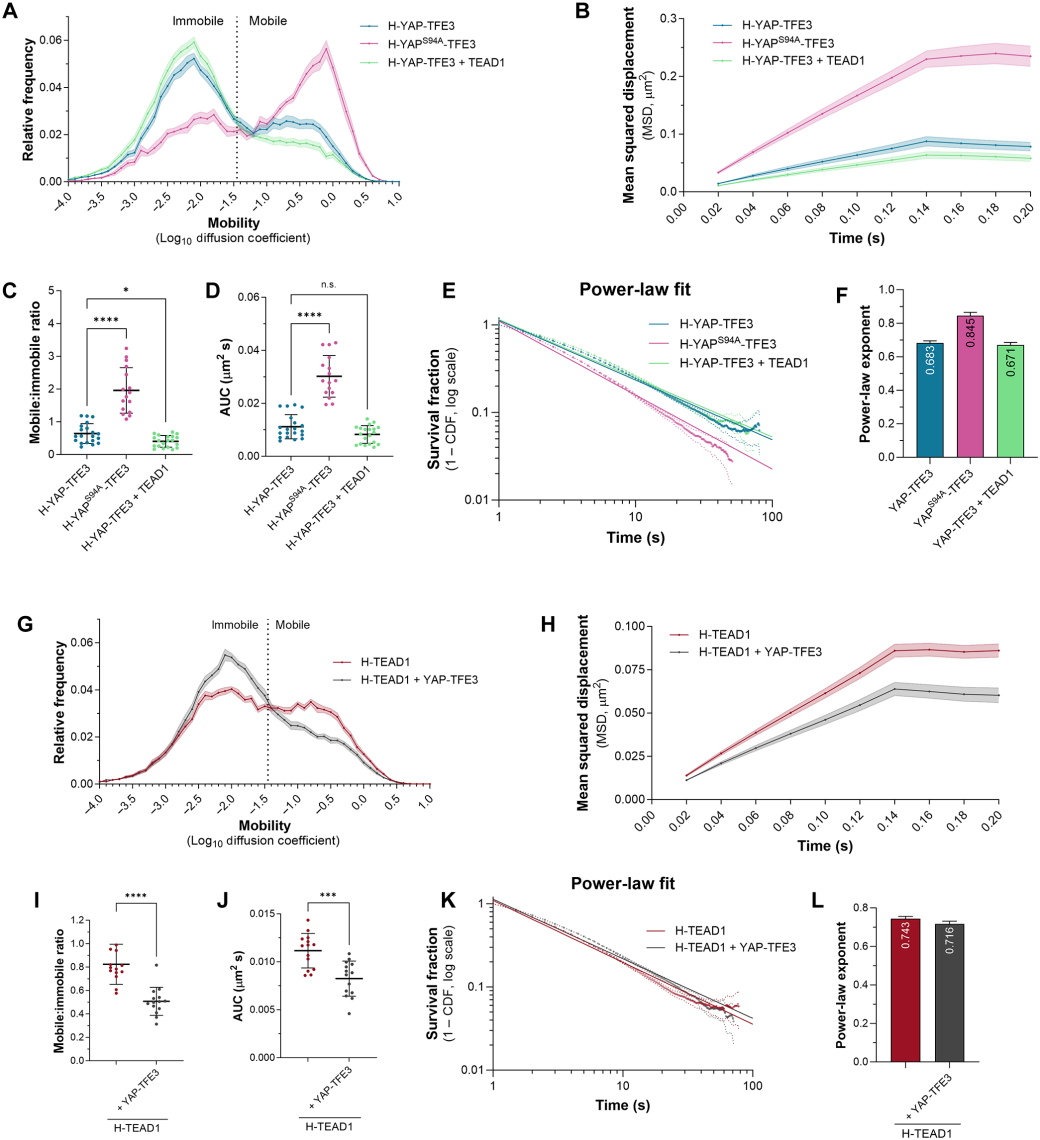
The nuclear behavior of YAP-TFE3 differs substantially from YAP. (**A**) Chart of relative frequency of molecule mobilities (log_10_ diffusion coefficient) of YAP-TFE3 (blue), YAP^S94A^-TFE3 (pink), and YAP-TFE3 with overexpressed TEAD1 (green) in HeLa cell nuclei over time (42,829 trajectories, 20 cells; 34,229 trajectories, 16 cells; and 71,291 trajectories, 20 cells); means ± SEM. (**B**) Chart showing MSD (square micrometers) of YAP-TFE3, YAP^S94A^-TFE3, and YAP-TFE3 with overexpressed TEAD1; means ± SEM. (**C** and **D**) Charts of mobile-to-immobile ratio (C) or AUC (D) (square micrometers per second) of YAP-TFE3, YAP^S94A^-TFE3, or YAP-TFE3 with overexpressed TEAD; *n* = 20,16,20; means ± SD; *P < 0.05; ****P < 0.0001 (Brown-Forsythe and Welch ANOVA with Dunnett’s T3 multiple comparisons test); n.s., not significant. (**E**) Chart of photobleach-corrected survival distribution of YAP-TFE3, YAP^S94A^-TFE3, or YAP-TFE3 with overexpressed TEAD1 (*n* = 167,283 trajectories, 27 cells; 104,009 trajectories, 19 cells; 86,983 trajectories, 20 cells), with power-law fits (solid lines). Dotted lines are 99% CI. (**F**) Chart of power-law exponents of YAP-TFE3, YAP^S94A^-TFE3, or YAP-TFE3 with overexpressed TEAD1, with 95% CI. (**G**) Chart of relative frequency of molecule mobilities (log_10_ diffusion coefficient) of TEAD1 ± YAP-TFE3-GFP (46,400 or 50,235 trajectories, 14 cells each); means ± SEM. (**H**) Chart of MSD (square micrometers) of TEAD1 molecules ± YAP-TFE3-GFP; means ± SEM. (**I** and **J**) Charts of mobile-to-immobile ratio (I) or AUC (J) (square micrometers per second) of TEAD1 molecules ± YAP-TFE3-GFP; means ± SD; ***P < 0.001; ****P < 0.0001 (Brown-Forsythe and Welch ANOVA with Dunnett’s T3 multiple comparisons test); *n* = 14 cells each. (**K**) Chart of photobleach-corrected survival distribution of Halo-TEAD1 molecules ± YAP-TFE3-GFP (121,486 trajectories, 16 cells; and 102,439 trajectories, 15 cells), with power-law fits (solid lines). (**L**) Chart of power-law exponents of TEAD1 molecules ± YAP-TFE3-GFP, with 95% CI.

Multiple Hippo pathway genes are mutated in different human cancers, including YAP and TAZ, which, following chromosomal translocations, can fuse with different transcription factors (*40*). One such fusion protein, YAP-TFE3, occurs in 10 to 20% of epithelioid hemangioendotheliomas (*41*), a vascular tumor that is caused by fusion of the N terminus of either YAP or TAZ with TFE3 or CAMTA1, respectively (*40*). To investigate whether the biophysical behavior of cancer-associated YAP fusion proteins differs from YAP, we performed SMT of HaloTagged YAP-TFE3. Fast tracking revealed that, like YAP and TEAD1, YAP-TFE3 exhibits both mobile and immobile behavior but is less mobile than either YAP or TEAD1 (Fig. 7, compare to Figs. 2 and 3). Multiple independent studies have revealed that YAP-TFE3 drives oncogenic transcription at least, in part, through TEADs. Therefore, to investigate the relative importance of TEAD binding for YAP-TFE3 nuclear behavior, we imaged a version of YAP-TFE3 that cannot interact with TEADs (Halo-YAP^S94A^-TFE3). YAP^S94A^-TFE3 was substantially more mobile than YAP-TFE3 (Fig. 7, A to D), indicating that TEADs are a major determinant of YAP-TFE3 nuclear behavior, as they are for YAP. This was further confirmed by the finding that TEAD1 overexpression further decreased the mobility of YAP-TFE3 (Fig. 7, A to D). Additionally, TEAD1 mobility was notably reduced by YAP-TFE3 overexpression (Fig. 7, G to J). Therefore, these studies reveal that cancer-associated alleles of YAP transform the nuclear dynamics of the wildtype transcription regulatory protein.

To investigate YAP-TFE3 DNA binding, we performed slow SMT. The DNA binding times of YAP-TFE3 were longer than overexpressed YAP or TEAD1, as revealed by a power-law exponent of 0.683 ± 0.012 (Fig. 7F, compare to Figs. 3I and 7J). TEADs also had an impact on YAP-TFE3 DNA binding; YAP^S94A^-TFE3 had a power-law exponent of 0.845 ± 0.019, indicating that association with TEADs facilitate more stable DNA contacts. However, TEAD1 overexpression did not substantially alter YAP-TFE3’s power-law exponent (0.671 ± 0.015; Fig. 7F). Consistent with the finding that TEAD’s influence YAP-TFE3’s DNA residence times, biexponential fitting showed that YAP^S94A^-TFE3’s long and short dwell times were reduced by more than half relative to YAP-TFE3 (YAP-TFE3, 54 and 3.8 s; and YAP^S94A^-TFE3, 21.4 and 2.2 s; fig. S10, B and C). Notably, disabling YAP-TFE3’s ability to bind TEADs did not prevent all immobile behavior of YAP-TFE3, presumably because TFE3 also has a DNA binding domain, and we previously found using genomics experiments that YAP-TFE3 binds the genome via both TEADs and TFE3 (*59*). Despite TEAD1 becoming less mobile in the presence of YAP-TFE3, slow SMT indicated no substantial changes to its DNA dwell times, as determined by the power-law fit (Fig. 7, K and L).

## DISCUSSION

Using advanced microscopy techniques, we revealed insights into the molecular mobility and DNA binding properties of the key transcription pair, YAP and TEADs, and how these features are regulated by Hippo pathway signaling. We found that both YAP and TEAD1 exhibit mobile and immobile behavior in the nucleus, with YAP being more mobile than TEAD1. YAP’s mobility was greatly affected by its ability to form a physical complex with TEADs, while YAP only had a minor influence on TEAD1 mobility. Both proteins bound chromatin across broad timescales from fractions of seconds through to minutes, with YAP generally having shorter DNA dwell times than TEAD1, which suggests three possibilities: (i) TEAD resides on DNA and YAP binds it there, and they unbind DNA simultaneously; (ii) YAP and TEAD first form a heterodimer in the nucleoplasm and bind DNA together, and then YAP disengages from TEAD before TEAD unbinds DNA; or (iii) a combination of these two possibilities. Our finding that YAP diffuses throughout the nucleus approximately three times faster than TEAD1 argues in favor of the first possibility, i.e., that YAP and TEAD1 predominantly form a heterodimer on DNA, rather than diffusing throughout the nucleus as a pre-formed heterodimer. This is further supported by the recent finding that the *Drosophila* orthologs of YAP and TEADs, Yorkie and Scalloped, diffuse at almost the same rates in nuclei of *Drosophila* tissues as YAP and TEAD1 (*37*). To explore this definitively, one would have to simultaneously track both single YAP and TEAD molecules. This is currently very technically challenging as SMT relies on a very low percentage of a given protein pool being labelled, meaning the frequency of capturing YAP/TEAD heterodimers would be very low.

SMT microscopy also revealed an important mechanism by which the Hippo pathway controls transcription, i.e., Hippo signaling normally limits the time that both YAP and TEAD1 bind to chromatin. Coupled with recent observations in *Drosophila* where overexpression of the Yorkie co-activator extended the DNA dwell times of Scalloped (*37*), this suggests that, in addition to regulating the nucleocytoplasmic distribution of YAP, the Hippo pathway controls transcription by regulating YAP/TEAD chromatin dwell time. The fact that similar discoveries have been made in both *Drosophila* and human cells further underscores how key features of this ancient signaling pathway have been conserved throughout evolution (*60*, *61*). We made these observations by acutely perturbing Hippo signaling using chemical inhibitors of the LATS1/2 kinases, as well as with a cell coculture model that recapitulates key features of the preimplantation mammalian embryo, i.e., that Hippo signaling is high in the inner cell mass, and low in the trophectoderm. In recent years, multiple groups have developed in vitro models of the human blastocyst, which form 3D embryo-like structures (*54*, *62*). Despite their utility for studying features of early human life, the 3D structures they form present challenges for some microscopy modalities such as HILO, which is limited by the distance of cell nuclei to the microscope objective. Given the advantages provided by the 2D iPSC/iTSC coculture model for live SMT microscopy described here, this model could offer a way to study the dynamics of additional transcription factors and signaling proteins that drive cell fate choices in early embryos.

Certain cancers are driven by oncogenic fusions with Hippo pathway transcription regulators, i.e., YAP, TAZ, or TEADs (*40*). Our high-resolution imaging studies showed that one such cancer fusion, YAP-TFE3, is less mobile and binds DNA longer than either YAP or TEAD1 and, like YAP, its nuclear behavior is highly dependent on TEADs. SMT revealed that TFE3 confers some DNA binding ability to YAP-TFE3, which is consistent with published genomic and functional studies (*59*, *63*). YAP-TFE3 also slowed TEAD1’s nuclear mobility, indicating that it may promote TEAD DNA target search and participate in cooperative DNA binding, for example, by the DNA binding domains of both TEADs and TFE3. This suggests that other oncogenic transcription factor fusion proteins also have nuclear behavior and DNA binding properties that differ from their endogenous proteins and influence the DNA binding kinetics of their interaction partners, although this remains to be tested.

During our studies, we noted that the chromatin binding times of both YAP and TEAD1 occurred along a broad continuum, with a predominance of short binding events occurring for less than a second, and rarer long binding events occurring for up to a minute or more. Therefore, we examined whether this could be explained by YAP and TEAD1 binding chromatin on different timescales in discrete nuclear sub-domains, paying close attention to nuclear condensates, given both that YAP and TAZ form condensates that have been linked to transcription activation (*33*–*36*). TEAD1 was less mobile, and its dwell times were substantially higher in condensates than other regions of the nucleus, again consistent with the notion that extended YAP/TEAD chromatin association correlates with active transcription. In support of this, both a hyperactive YAP protein and the BRD4 and MED1 proteins, which activate transcription with YAP (*56*, *57*), were enriched in TEAD condensates. Furthermore, acute disruption of the YAP/TEAD protein-protein interaction using a small molecule (IAG933) (*58*), caused the rapid eviction of YAP, BRD4, and MED1 from TEAD condensates. By contrast, acute IAG933 treatment actually increased the size and number of TEAD1 condensates and caused TEAD1 DNA dwell times to increase in nuclear condensates. At first glance, this seems at odds with our observations that longer YAP/TEAD DNA binding times are associated with transcription activation and that TEAD1^Y421H^ (which cannot bind YAP) exhibits similar nuclear behavior to wild-type TEAD1. However, it is important to note that TEAD is a default repressor of transcription (*17*, *18*), and that chromatin binding by TEADs and the VGLL4 transcription corepressor is favored upon IAG933 treatment (*58*). Thus, we postulate that the extended TEAD1 DNA binding times observed in nuclear condensates following acute IAG933 treatment reflect the transcription repression function of TEAD1/VGLL4. For example, TEAD1 might bind longer to chromatin upon acute IAG933 treatment because the TEAD1/VGLL4 complex recruits chromatin regulatory proteins to establish a repressive chromatin environment. In line with this idea, examination of nuclear behavior of TEAD1 that cannot bind YAP in a non-acute setting (i.e., TEAD1^Y421H^) revealed that it bound DNA for shorter times than wildtype TEAD1. In future studies, it will be interesting to determine whether this is a general feature of transcription factors that, like TEADs, have dual activation/repression roles depending on their cofactor binding.

Another question raised by our and other studies that have revealed that transcription factor–DNA binding coincides with transcription activation is how exactly does a transcription cofactor influence DNA binding time of its cognate transcription factor? Several possibilities exist; for example, a transcription cofactor could allosterically increase the affinity of transcription factors for their cognate DNA binding motifs. Alternatively, a recent study showed that nascent mRNA can form a physical complex with transcription factors and stabilize their interactions with DNA (*64*). Additionally, this phenomenon could be explained by proteins that can form phase separated condensates through intrinsically disordered regions (*65*). As detailed, here and previously (*34*), such proteins exhibit reduced mobility inside condensates, which may promote DNA target search and could theoretically extend the chromatin binding time of their partner proteins.

### Limitations

The main microscopy approach used in this study, SMT, is limited by photobleaching, even with the use of photostable fluorescent dyes and gentle imaging modalities like HILO (*43*). Bleaching will have a disproportionate impact on long-lived DNA binding events; one way to alleviate this is with the use of photobleaching correction methods (*45*), as used here, but the longest DNA binding events are likely to still be underestimated. Further, while we studied protein mobility in scenarios that are known to robustly modulate Hippo-regulated transcription, we did not assess transcription of individual gene loci in real time and binding of individual YAP and TEAD1 molecules specifically to them. Finally, given the very high degree of homology and similar behavior in numerous functional studies (*66*), we chose to study only one TEAD family protein (i.e., TEAD1, but not TEAD2, TEAD3, or TEAD4). It is unlikely but formally possible that TEAD2, TEAD3, and/or TEAD4 exhibit different biophysical behaviors than TEAD1.

## MATERIALS AND METHODS

### Plasmids

All HaloTag plasmids (both N- and C-terminally tagged) were created by cloning cDNAs into either a pReceiver-M49-HaloTag plasmid (for transient transfections) or a plenti-DEST-CMV-Neo plasmid for doxycycline-inducible stably expressed plasmids. All GFP or RFP657 plasmids were created by cloning cDNAs into pEGFP-N1, pEGFP-C1, or pLenti-CMV-RFP657. The HA-TEAD1 plasmid created by cloning the TEAD1 cDNA into plenti-DEST-CMV-Neo. All mutations were engineered using polymerase chain reaction–based mutagenesis.

### Cell culture

HeLa and human embryonic kidney (HEK) 293T cells were cultured in Dulbecco’s modified Eagle’s medium (DMEM; Thermo Fisher Scientific) supplemented with 10% fetal bovine serum (FBS; HyClone, SH30396.03). MCF10A cells were cultured in DMEM + F12 supplemented with horse serum (Life technology), epidermal growth factor human (Sigma-Aldrich, SRP3027-500UG), hydrocortisone (Sigma-Aldrich, H0135-1MG), cholera toxin (Sigma-Aldrich, C8052-2MG), and Insulin (Actrapid Penfill, 100 U/ml). iPSCs were reprogrammed and maintained in a feeder-free E8 medium (Thermo Fisher Scientific, A1517001) with vitronectin (VTN-N; Thermo Fisher Scientific, A14700) as published (*67*). Cells were cultured in 37°C, 5% O_2_, and 5% CO_2_, and medium was changed daily. Cells were passaged every 3 to 5 days with 0.5 mM EDTA (Thermo Fisher Scientific, 15575-038). iTSCs were reprogrammed and maintained in collagen IV (Sigma-Aldrich, C5533)–coated plates in the following medium, as published (*67*). DMEM/F12 GlutaMAX (Thermo Fisher Scientific, 10565018) with 0.3% (w/v) bovine serum albumin (Sigma-Aldrich, A9576), 0.2% (v/v) FBS (HyClone, SH30071.03), 1% (v/v) Insulin-Transferrin-Selenium-Ethanolamine (ITS-X) supplement (Thermo Fisher Scientific, 51500056), 0.1 mM 2-mercaptoethanol (Thermo Fisher Scientific, 21985023), 0.5% (v/v) penicillin/streptomycin (Thermo Fisher Scientific, 15140122), l-ascorbic acid (1.5 μg/ml; Sigma-Aldrich, A4544), 5 μM Y27632 (Selleckchem, S1049), 2 μM CHIR99021 (Sigma-Aldrich, SML1046), 0.5 μM A83-01 (Sigma-Aldrich, SML0788), 1 μM SB431542 (Selleckchem, S1067), epidermal growth factor (50 ng/ml; Peprotech, AF-100-15), and 0.8 mM valproic acid (Sigma-Aldrich, P4543). Cells were cultured in 37°C and 5% CO_2_, and media were changed every other day. Cells were passaged every 4 to 5 days with TrypLE express (Thermo Fisher Scientific, 12604021). Novartis provided IAG933 for this research.

### Viral transduction of cell lines

HEK293T cells were transfected using either polyethyleneimine transfection or LTX methods to generate lentiviral particles to transduce Halo-YAP/TEAD1 expression vectors. A lentiviral packaging mixture was prepared by combining OptiMEM, psPAX2, pMD2.G, and the lentiviral expression vector. iPSC and iTSC were plated at a density of 5 × 10^4^ cells per well in a 12-well plate; after incubation at 37°C for 24 hours, transduction was performed with a multiplicity of infection (the ratio of infectious virions to cells) of 1 and 5, respectively, at a final volume of 0.5 ml per well with culture medium in the presence of polybrene transfection reagent (1:1700; TR-1003-G, EMD Millipore). The culture medium was replaced with 1 ml of culture medium every 24 hours. After 72 hours, transduced cells were passaged or sorted on the BD influx system flow cytometer. GFP-positive cells from the cultured iPSC Halo-YAP, iPSC Halo-TEAD1, iTSC Halo-YAP, and iTSC Halo-TEAD1 were collected and cultured.

### iPSC/iTSC coculture

For Halo-YAP or Halo-TEAD1, iTSCs were seeded at a density of 30 × 10^4^ cells per dish in the imaging dishes (ibidi, 81156) coated with collagen IV. After 48 hours, media were removed, and 40 × 10^4^ of iPSCs were added in E8 medium with Y-27632 (10 μM), respectively. Doxycycline (2 μg/ml, Sigma-Aldrich, 33429-100MG-R) was also added, and cells were cultured in 37°C and 5% CO_2_ for 24 hours before imaging. Phenol red–free DMEM/F12 (Thermo Fisher Scientific, 11039-021) supplemented with E8 supplement was used during image.

### Immunoblotting

Whole-cell lysates were prepared in radioimmunoprecipitation assay buffer and subjected to SDS–polyacrylamide gel electrophoresis. Proteins were transferred to polyvinylidene difluoride membranes (Millipore) and probed with primary antibodies specific for the following proteins: YAP (Cell Signaling Technology, 4912), phospho-YAP (Ser^127^; Cell Signaling Technology, 4911), TEAD1 (BD Biosciences, 610922), or tubulin (Sigma-Aldrich, T9026), followed by detection with secondary antibodies and chemiluminescence visualization.

### Immunofluorescence

iTSCs and iPSCs fixed in 4% paraformaldehyde were immunostained with mouse anti-YAP (Abnova, H00010413-M01) at 1:100 dilution. HeLa cells fixed in ice cold 100% methanol were immunostained with either rabbit anti-BRD4 (Abcam, 128874) at 1:200 dilution or rabbit anti-MED1 (Bethyl Laboratories, A300-793A) at 1:500 dilution. Secondary antibodies conjugated to either Alexa Fluor 568 or Alexa Fluor 647 (Invitrogen) were used at a concentration of 1:500. Hoechst 33342 (1 μg/ml) was used to stain nuclei.

### SMT microscopy

To obtain optimal single-molecule staining of Halo-YAP or Halo-TEAD1, cells were incubated with ~2 to 5 nM Halo JF549 ligand (Promega) in their media for ~15 to 30 min before imaging. In experiments to label all Halo-tagged molecules, Halo JF549 or Halo JFX650 ligand was applied at a concentration of 100 nM (e.g., Fig. 6 and fig. S8). In most experiments, DNA was simultaneously stained using Hoechst 33342 (NucBlue Live ReadyProbes Reagent, Invitrogen). Cells were maintained at 37°C with 5% CO_2_ on the microscope using a Tokai Hit incubation system. SMT was carried out on a Zeiss Elyra microscope using a Zeiss α Plan-Apochromat [100×/1.46 numerical aperture (NA) OIL DIC VIS] objective. The HILO imaging modality was used in combination with a high-power TIRF field (TIRF_HP) and an additional 1.6× OptoVar Lens. TIRF angles of 57° (iPSCs/iTSCs), 58° (MCF10A cells), or 59° (HeLa cells) were used to optimally section the nuclear plane with HILO. Halo JF549 ligand was excited using a 200-mW 561-nm laser using either 10% (Fast SMT) or 5% (Slow SMT) laser power with emission light passed through 570- to 620-nm band-pass filter and detected using an Andor iXon 897 EMCCD camera. Hoechst 33342 was visualized using a 50-mW, 405-nm laser with emission light passed through a 420- to 480-nm band-pass filter to the camera. When bulk Halo-tagged TEAD1 was imaged, Halo-JFX650 was excited using a 150-mW, 642-nm laser and emission light passed through a 650-nm long-pass filter to the camera. The microscopy parameters, lasers, and cameras were controlled through Zen 2012 SP5 (Black, version 14.0.0.0). Using these parameters, we performed two different acquisition techniques: fast SMT, which uses a 20-ms acquisition speed to acquire 6000 frames without intervals, and slow SMT, which uses a 500-ms acquisition speed to acquire 500 frames without intervals. The only exception to this was our analysis of TEAD1 condensates (Fig. 6 and fig. S8). In this case, 3000 frames were acquired for fast SMT and 250 frames for slow SMT, before and after IAG933 treatment, in the same cells.

### Fast SMT

Analysis was performed as in (*68*). Briefly, raw fast (20 ms) SMT data were analyzed using the PALMTracer plugin for Metamorph (*44*, *69*). PALMTracer was used to localize and track molecules to obtain their trajectories and to calculate the MSD and diffusion coefficient (*D*) for each trajectory. For molecule localization, we used a watershed of size 6. To reduce nonspecific background and to reduce the likelihood of mistracking, trajectories were filtered on the basis of a minimum length of 8, a maximum length of 1000, and a maximum travel distance of 5 μm. For visualization of trajectories, a zoom of 8 with a fixed intensity and size of 1 was used. A spatial calibration of 100 nm and a temporal calibration of 20 ms was used.

Analysis files produced by PALMTracer were then used as input for AutoAnalysis_SPT software (https://github.com/QBI-Software/AutoAnalysis_SPT/wiki). AutoAnalysis_SPT compiles the results obtained for each cell to obtain the average MSD, calculates the average AUC of the MSD for each cell, generates a histogram showing the distribution of the different log_10_ diffusion coefficients, and calculates the mobile-to-immobile ratio for each cell. Here, we used 10 MSD points, with a time interval of 0.02 s (20-ms acquisition time), and included trajectories with a minimum log_10_ diffusion coefficient of −5 and a maximum log_10_ diffusion coefficient of 1. For the log_10_ diffusion coefficient histogram, a bin width of 0.1 and a mobile-to-immobile threshold of −1.45 (~0.035 μm^2^/s) were selected on the basis of previous studies (*68*).

### Slow SMT

Images were acquired with an exposure time of 500 ms such that fast-moving molecules were blurred out and only immobile and DNA-bound molecules were observed. Single-molecule localization and tracking were performed using SLIMfast for MATLAB (version 2015a), as in (*42*, *68*, *70*, *71*). SLIMfast uses a modified version of the multiple-target tracing algorithm (*72*). SLIMfast batch processing was performed using an error rate of 10^−7^, a detection box of 7 pixels, maximum number of iterations of 50, a termination tolerance of 10^−2^, a maximum position refinement of 1.5 pixels, an NA of 1.46, a point spread function (PSF) scaling factor of 1.35, 20.2 counts per photon, an emission of 590 nm, a lag time of 500 ms, and a pixel size of 100 nm. Trajectories were filtered with the maximum expected diffusion coefficient of 0.1 μm^2^/s. Dwell time data were compiled for each condition, and a frequency distribution was generated using GraphPad Prism. Dwell time data were subsequently photobleach corrected and analyzed in MATLAB (version 2024a). To exclude the effect of the noisy tail of the survival distribution, time points with fewer than 30 cumulative events were excluded from fitting, except in the case of Fig. 6, where all values were included in the fitting. As described in (*37*, *45*), the photobleaching rate of Halo JF549 ligand was determined using SMT of Halo-H2B, expressed in HeLa cells, and acquired using different TIRF angles to reflect different experimental settings. Survival distributions were fit to bi- or triple-exponential functions, and the best fit was determined using BIC. The smallest exponential component, corresponding to the bleaching rate of JF549, was used for subsequent correction of slow tracking SMT data. Bleach-corrected survival distributions derived from tracking data of Halo-tagged proteins were fit to a biexponential fit or a power-law fit.

### Confocal microscopy

Live confocal microscopy of Halo-TEAD1 and YAP-GFP (Fig. 6) was performed using a Zeiss Elyra LSM780 microscope, equipped with a Zeiss C-Apochromat (63×/1.2 NA W Korr UV-VIS-IR) objective, 488-nm (GFP) or 561-nm (Halo-JF549) lasers, and a spectral GaAsP detector with two flanking PMT’s (detection windows used were 490 to 553 nm for GFP and 562 to 641 nm for Halo-JF549). Cells were maintained at 37°C with 5% CO_2_ on the microscope using a Tokai Hit incubation system. For LATSi experiments (Fig. 4), cells were first treated with either DMSO or 3 μM LATSi (*52*) for 2 hours before imaging. For IAG933 experiments (Fig. 6), a 10× solution of IAG933 (*58*) in imaging medium was added to cells (to a working concentration of 3 μM) during microscopy and immediately imaged.

### Raster image correlation spectroscopy

All RICS experiments were performed on an Olympus FV3000 laser scanning microscope. A 60× water immersion objective 1.2 NA was used for all experiments, and live HeLa or MCF10A cells were imaged at 37°C in 5% CO_2_. For RICS experiments on Halo-YAP or Halo-TEAD1 (Fig. 1F and fig. S5B), cells were stained for 15 min with 1 μM JF549 HaloTag dye to label all molecules, where JF549 was excited by a solid-state laser diode operating at 561 nm. The JF549 emission was collected through a 550-nm long-pass filter by an external photomultiplier detector (H7422P-40 of Hamamatsu) fitted with a 620/50-nm bandwidth filter. A 100-frame scan acquisition of the JF549 signal was collected by selecting a region of interest within a nucleus at zoom 20, with a 1 Airy unit pinhole size which for a 256 × 256-pixel frame size resulted in a pixel size of 41 nm. The pixel dwell time was set to 12.5 μs, which resulted in a line time of 4.313 ms and a frame time of 1.108 s. RICS analysis was carried out using SimFCS software (Globals), using a 10-frame moving average subtraction. This involved application of the RICS function to each time series acquisition and extraction of the apparent diffusion coefficient (*D*) by fitting each resulting 3D RICS profile to a one-component diffusion model.

### Statistical analyses

GraphPad Prism (version 10.1.2) was used to generate graphs and to perform statistical analyses.
